# Subpopulations of exosomes purified via different exosomal markers carry different microRNA contents

**DOI:** 10.7150/ijms.52768

**Published:** 2021-01-01

**Authors:** Shao-Chun Wu, Pao-Jen Kuo, Cheng-Shyuan Rau, Yi-Chan Wu, Chia-Jung Wu, Tsu-Hsiang Lu, Chia-Wei Lin, Chia-Wen Tsai, Ching-Hua Hsieh

**Affiliations:** 1 Department of Anesthesiology, Kaohsiung Chang Gung Memorial Hospital and Chang Gung University College of Medicine, Taiwan; 2 Department of Plastic Surgery, Kaohsiung Chang Gung Memorial Hospital and Chang Gung University College of Medicine, Taiwan; 3 Department of Neurosurgery, Kaohsiung Chang Gung Memorial Hospital and Chang Gung University College of Medicine, Taiwan

**Keywords:** Biomarkers, Exosome, MicroRNA, Next-generation sequencing, Subpopulation, Surface markers

## Abstract

The heterogeneity of exosome populations presents a great challenge to their study. The current study was designed to investigate the potential heterogeneity miRNA contents in circulating exosomes purified via different exosomal markers. In this study, exosomes from the serum of C57BL/6 mice after cecum ligation and perforation (CLP) or sham operation were isolated by precipitation using ExoQuick-TC and affinity purified with anti-Rab5b, anti-CD9, anti-CD31, and anti-CD44 antibodies using the Exo-Flow Exosome Capture kit to collect exosome subpopulations. RNA extracted from the exosomes isolated by ExoQuick-TC were profiled by next-generation sequencing (NGS). Real-time quantitative reverse transcription polymerase chain reaction (RT-qPCR) was also employed to determine the expression profiles of four representative exosomal miRNAs (mmu-miR-486-5p, mmu-miR-10a-5p, mmu-miR-143-3p, and mmu-miR-25-3p) selected from the NGS analysis. The results revealed that the expression patterns of these miRNAs in exosomes isolated by ExoQuick-TC as determined by RT-qPCR and NGS were similar, showing upregulation of mmu-miR-10a-5p and mmu-miR-143-3p but downregulation of mmu-miR-25-3p and mmu-miR-486-5p following CLP when compared to the levels in exosomes from sham control mice. However, their expression levels in the antibody-captured exosome subpopulations varied. The miRNAs in the exosomes captured by anti-Rab5b or anti-CD9 antibodies were more similar to those isolated by ExoQuick-TC than to those captured by anti-CD44 antibodies. However, there were no significant differences in these four miRNAs in CD31-captured exosomes. This study demonstrated that purification with different exosomal markers allows the collection of different exosome subpopulations with various miRNA contents. The results of this study demonstrate the heterogeneity of circulating exosomes and suggest the importance of stratifying exosome subpopulations when using circulating exosomes as biomarkers or investigating exosome function. In addition, this study also emphasized the necessity of using a consistent exosome marker across different samples as detecting biomarkers.

## Introduction

Exosomes are a type of extracellular vesicle (EV) secreted by a variety of cells that are composed of lipid bilayers and carry different molecules, including DNA, mRNAs, microRNAs (miRNAs), non-coding RNAs, and proteins 1. Exosomes have been suggested to mediate intercellular communication 2 fundamental roles in numerous physiological and pathological processes 3, 4.

The results of some studies have indicated that most miRNAs are expressed at similar levels in cells and exosomes 5, 6, thus leading to the proposition that exosomes originating from cancer cells could be used as biomarkers, as they contain the same miRNA contents as their parent cells 6, 7. While exosomes share a set of common contents, their composition may be strongly dependent on their parent cells and their physiopathological conditions 8, 9. Cells release distinct exosome subpopulations with unique compositions 9. It also is expected that exosomes are delivered to recipient cells through surface proteins that have affinity for receptors on the recipient cells 10, and thus they elicit differential effects 9. Surface proteins that have been commonly used for the identification or purification of exosomes include tetraspanins (CD9, CD63, CD81, and CD82), proteins involved in the biogenesis of multivesicular bodies (TSG101 and Alix), the membrane trafficking protein Rab5b, and the membrane transport and fusion protein flotillin 7, 11-13. Although exosomes can be purified by size-exclusion chromatography, density gradient ultracentrifugation, or ultrafiltration, fractionation of their subpopulations is dependent on affinity techniques 14-16. Proteomic analyses have shown wide variation in the cellular expression of different tetraspanins in cells 17, and different exosomal subpopulations have been isolated by differential separation via immuno-isolation using either CD63, CD81, or CD9 17.

Studies on the enrichment of miRNAs in EVs suggest that miRNAs may be selectively packaged into EVs 18, and a study of cultured cells confirmed differential packaging of miRNAs into distinct subpopulations of EVs 19, with functional transfer of the miRNAs via EVs 20, 21. However, it is still unclear if the miRNA cargo in different exosome subpopulations isolated by affinity techniques carry more of certain miRNAs than others. Under the hypothesis that a profound critical illness with diverse pathophysiologic responses, like sepsis, may occur with secreted diverse exosome subpopulations for subsequent observation and experimental study, the present study was designed to investigate the miRNA profiles of circulating exosomes from septic mice by affinity techniques and the exosomes subpopulations captured by affinity purification using antibodies against Rab5b, CD9, CD31, and CD44. These findings revealed the existence of exosome subpopulations with unique miRNA contents.

## Methods

### Animal models of sepsis

Male C57BL/6 mice (BioLasco, Taipei, Taiwan) were housed in a specific-pathogen-free (SPF) facility, which is accredited by the Association for Assessment and Accreditation of Laboratory Animal Care International (AAALAC). Mid-grade sepsis was induced in the animal models by cecum ligation and perforation (CLP) 6. Briefly, mice were anesthetized with a combination of 0.1 mg/g ketamine and 0.01 mg/g xylazine. Via midline abdominal incision, the cecum was mobilized and ligated in the middle of the cecum, below the ileocecal valve, punctured once using a 21-G needle, and a small stool sample was squeezed out of the cecum to induce polymicrobial peritonitis. The abdominal wall was closed in two layers. Sham-operated mice underwent the same procedure, including opening of the peritoneum and exposing the bowel, but without ligation and needle perforation of the cecum. After surgery, the mice were resuscitated by subcutaneous injection of pre-warmed (37°C) normal saline (at 5 mL per 100 g of body weight). Total fifty-four mice were used in this study. All animal protocols were approved by the Institutional Animal Care and Use Committee of Chang Gung Memorial Hospital. All surgical procedures, including analgesia, were performed according to national and institutional guidelines.

### Blood sample collection and exosome isolation

At 16 h after surgery, 0.5 mL of whole blood were collected from each sham-operated and CLP-treated mouse into tubes containing anticoagulant. After incubation at room temperature for 15 min, the samples were centrifuged at 3,000 × *g* for 10 min. The white blood cells were slowly removed from the corresponding layers, and serum samples were extracted. Exosomes were isolated with the exosome isolation reagent, ExoQuick-TC (System Biosciences, Palo Alto, CA, USA). Briefly, the supernatants were transferred to sterile tubes containing 63 μL of ExoQuick-TC Precipitation Solution (System Biosciences), mixed, and incubated for at least 12 h at 4°C. After incubation, the samples were centrifuged at 1,500 × *g* for 30 min at 4°C. The white pellet containing exosomes was resuspended in 500 μL of buffer.

### Purification of exosome subpopulations

Isolated exosomes were further purified using the immune-affinity Exo-Flow Exosome Capture kit (System Biosciences). Briefly, 40 µL of biotinylated capture antibodies (Rab5b, CD9, CD31, and CD44) were conjugated to 10 µL of Exo-Flow 9.1 µm streptavidin-conjugated magnetic beads on ice for 2 h to allow for the efficient capture of exosomes expressing these surface markers. Next, the samples were incubated on a rotating rack at 4°C overnight. To validate the isolation procedure, 200 µg of exosome-coated beads were stained with Exo-FITC exosome stain (System Bioscience) on ice for 2 h and then analyzed with a BD LSR II flow cytometer (BD Biosciences). Beads without the biotinylated capture antibodies were used as negative controls.

### Characterization of exosomes

#### Expression of exosomal surface markers

Expression of exosomal surface markers on the ExoQuick-isolated exosomes was detected by western blotting, in triplicate. Serum samples were used as a negative control. Exosome samples from the blood of CLP and sham mice were lysed, and the total proteins were separated by polyacrylamide gel electrophoresis (PAGE) and electrotransferred to polyvinylidene fluoride (PVDF) membranes (Millipore, Billerica, MA, USA). The membranes were blocked with 5% skim milk in PBS/Tween-20 and incubated with primary antibodies against CD9 (cat # ab92726, 1:1000; Abcam, Cambridge, MA, USA), TSG101 (cat # ab30871, 1:1000; Abcam), Flotillin-1 (cat # 18634, 1:1000; Cell Signaling Technology, Danvers, MA, USA), CD81 (cat # ab109201, 1:1000; Abcam), and Calnexin (cat # ab22595, 1:1000; Abcam) at 4°C overnight. The PVDF membranes were washed with 0.1% TBS/Tween 20 for 10 min, three times at room temperature and incubated with horseradish peroxidase (HRP)-conjugated secondary antibodies (cat # NA931; GE Healthcare Amersham, Piscataway, NJ, USA) for 2 h at room temperature, and the detected proteins were quantified using a FluorChem SP imaging system (Alpha Innotech, San Leandro, CA, USA).

#### Transmission electron microscope (TEM) analyses

Exosome samples in 10 µL amount were fixed with 2.5% glutaraldehyde for 2 h and added to a 200 mesh Formvar which was stabilized with carbon. The grids were stained with 2% uranyl acetate for 1 h. Samples were analyzed with a transmission electron microscope HT-7700 in 100 kV (Hitachi, Tokyo, Japan).

#### Dynamic light scattering (DLS) analysis

A Zetasizer Nano-ZS dynamic light scattering (DLS) system (Malvern, Montréal, QC, Canada) was used to measure the particle hydrodynamic diameter of the isolated exosomes. Each sample (100 mL) was loaded into an ultraviolet microcuvette (BRAND; Essex, CT, USA) at 4°C. The Brownian motion of a particle was measured by the fluctuations of scattered light intensity at a wavelength of 633 nm and a fixed angle of 173° to indicate the velocity distribution of particle movement in solution. The diameter of the exosomes was measured using the Stokes-Einstein equation to determine the particle's hydrodynamic radius. Each data point from each replicate represents an average of three measurements of 12-18 runs, which was set automatically. The average particle diameter was obtained from the peak of the Gaussian model fit to the particle distribution. The polydispersity index (PdI) was determined to reflect the width of the primary size distribution in solution 22.

#### RNA isolation

To detect the exosomal miRNAs, exosomes were eluted from magnetic beads by incubation in elution buffer for 2 h on a rotating rack. Total RNA from the exosomes was enriched using the SeraMir ExosomeRNA Amplification kit (System Bioscience). The purified RNA yield was determined by measuring the absorbance at 260 nm using an SSP-3000 Nanodrop spectrophotometer (Infinigen Biotech, City of Industry, CA, USA), and RNA quality was evaluated with an Agilent Bioanalyzer (2100; Agilent Technologies, Palo Alto, CA, USA).

#### Next-generation sequencing (NGS)

RNA samples from the circulating exosomes of three mice with CLP were pooled for next-generation sequencing (NGS). The exosomal RNA samples from three mice without CLP (sham) were pooled for use as a control. The RNA samples were sent to GeneTech Biotech Co., Ltd. (Taipei, Taiwan) for cloning. The miRNA population, with lengths of 15-30 nucleotides (nt), was passively eluted from polyacrylamide gels, precipitated with ethanol, and dissolved in water. Linkers were ligated to the small RNAs, and bar-coded cDNAs were prepared using the TruSeq Small RNA Sample Prep Kit (Illumina, San Diego, CA, USA) according to the manufacturer's instructions. Briefly, Adapters were ligated to the 3′ and 5′ ends of an aliquot (1 μg) of the pooled small RNAs. Then, adapter-ligated RNAs were reverse transcribed with SuperScript II Reverse Transcriptase (Invitrogen, Carlsbad, CA, USA) and amplified by polymerase chain reaction (PCR) in 15 cycles. The samples were indexed with barcodes of 15 variants of the reverse primers. A barcode was ligated directly to the miRNA to significantly reduce sample bias. Individual libraries were analyzed on a BioAnalyzer (Agilent Technologies) for the presence of linked cDNA, and 11 bar-coded libraries of the appropriate size (135-165 bp) were generated.

#### Quantification of miRNA expression

Real-time quantitative reverse transcription polymerase chain reaction (RT-qPCR) was employed to determine the expression profiles of selected representative exosomal miRNAs selected from the NGS analysis. The four miRNAs selected (mmu-miR-486-5p, mmu-miR-10a-5p, mmu-miR-143-3p, and mmu-miR-25-3p) were the most abundant miRNAs in the circulating exosomes of the mice following CLP, as shown by the NGS analysis. Each RNA sample was reverse transcribed to cDNA by using the TaqMan® MicroRNA Reverse Transcription Kit (Applied Biosystems, Foster City, CA, USA) according to the manufacturer's instructions. The PCR products were mixed with TaqMan Universal PCR Master Mix (No. UNG, PN 4324018, Applied Biosystems) and specific miRNA primers from the TaqMan MicroRNA Assays Kit (Applied Biosystems). As an internal control for the expression of each miRNA, 25 fmol of single-stranded cel-miR-39, synthesized by Invitrogen, was added. The RT-qPCR was run on a 7500 real-time PCR system (Applied Biosystems), and the relative expression levels were calculated in six samples and compared to those in the control samples. The expression of a given miRNA within the total exosomes isolated with ExoQuick-TC and the affinity-purified subpopulations captured with the Rab5b, CD9, CD31, and CD44 Exo-Flow Exosome Capture Kits was compared between mice with CLP or without (sham) (n = 6 for each subgroup). Prism 7 (GraphPad Software, San Diego, CA, USA) was used for statistical analysis, and differences were considered significant if the mean value differed from the control by more than two fold, with a p-value less than 0.05.

#### Functional annotation of the predicted targets of the differentially expressed miRNAs

The miRSystem (http://mirsystem.cgm.ntu.edu.tw/) was used for target prediction and functional annotation of the differentially expressed miRNAs within the total exosomes and exosome subpopulations captured by different surface markers. The miRSystem is a web-based system that is used to identify the biological functions/pathways regulated by miRNAs based on the functions of their predicted target genes by integrating seven miRNA target gene prediction databases (DIANA, miRanda, miRBridge, PicTar, PITA, rna22, and TargetScan) and two experimentally validated databases (TarBase and miRecords) 23. The analysis parameters in miRSystem were set as follows: (1) hit frequency = 5, (2) observed to expected (O/E) ratio = 2, (3) minimal size of genes annotated by ontology term for testing >50, and (4) matched pathways from the Kyoto Encyclopedia of Genes and Genomes (KEGG) database. The R statistical package (version 3.3.3) was used for hierarchical clustering of the annotated functions.

## Results

### Characterization of total exosomes and exosome subpopulations

**Figure 1:**
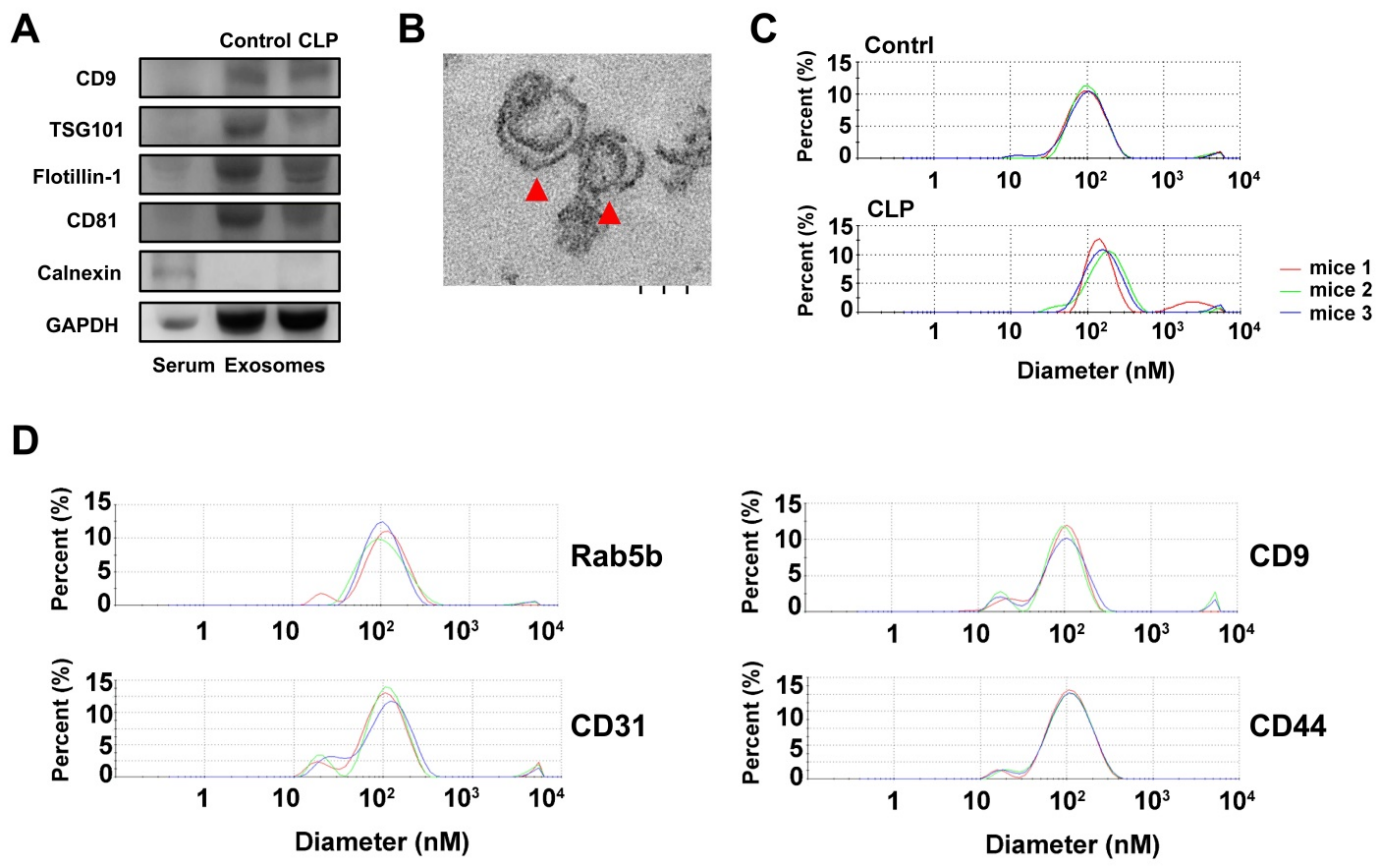
Characterization of exosomes isolated by ExoQuick-TC from mice with or without cecum ligation and perforation (CLP) by (A) western blotting for the exosomal surface markers CD9, TSG101, flotillin-1, CD81, and negative control marker Calnexin using serum samples as a control; (B) the morphology of exosomes as detected by transmission electron microscopy, red arrowheads indicate exosomes; and (C) the average size of the exosomes as quantified by dynamic light scattering analysis. (D) The average size of the exosomes quantified by dynamic light scattering analysis of those affinity-purified subpopulations captured with the Rab5b, CD9, CD31, and CD44 Exo-Flow Exosome Capture Kits.

**Figure 2:**
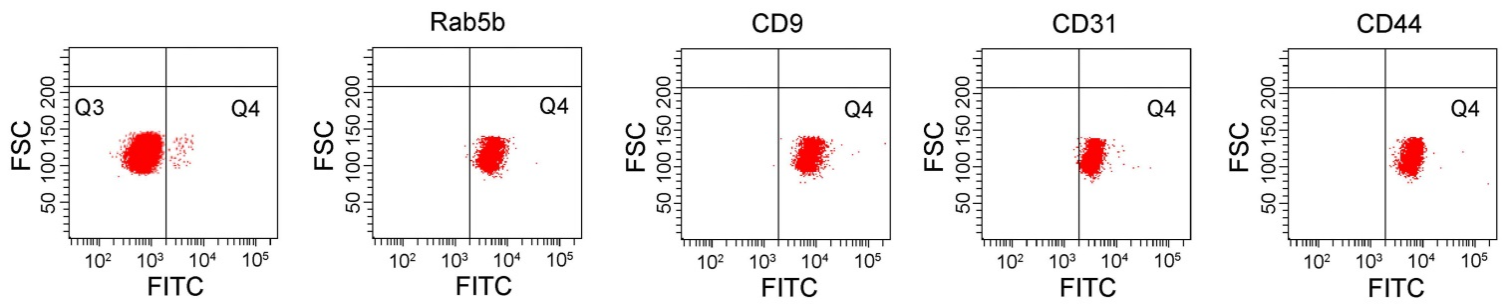
Isolated exosomes were further purified using the immune-affinity Exo-Flow Exosome Capture kit, detected by flow cytometry, and plot as forward-scattered light (FSC) versus FITC intensity in the bead flow separation for the various capture antibodies (Rab5b, CD9, CD31, and CD44) coupled with FITC staining. Beads without the biotinylated capture antibodies were used as negative controls.

To characterize the exosomes isolated by ExoQuick-TC, the expression of the positive exosomal surface markers CD9, TSG101, flotillin-1, CD81 and negative control markers calnexin were evaluated by western blotting for exosome samples from mice treated with CLP or without (sham), but not from serum samples (Figure 1A). The exosomes displayed a cup-shaped morphology with lipid bilayers and acceptable quality in terms of their size range and morphology as evaluated by TEM (Figure 1B). DLS analysis (Figure 1C) showed that, for mice treated with and without CLP, the average exosome size was 102.4 ± 15.2 nm and 119.0 ± 21.4 nm, respectively, and the PDI was approximately 0.28 and 0.41, respectively. The size distributions were single-peaked, with relatively good quality and even size distribution for those exosomes isolated by ExoQuick-TC and for those subsequently affinity-purified subpopulations captured with the Rab5b, CD9, CD31, and CD44 Exo-Flow Exosome Capture Kits (Figure 1D). In Figure 2, the bead flow separation data for the various capture antibodies coupled with FITC staining are shown as plots of forward-scattered light (FSC) versus FITC intensity. The addition of FITC to exosomes captured with antibodies against Rab5b, CD9, CD31, and CD44 resulted in increased FITC intensities when compared with exosomes that were not stained with FITC, indicating good separation of the different exosome subpopulations by the Exo-Flow Exosome Capture Kits.

### NGS analysis of miRNAs

The NGS analysis data are shown in Supplemental Table 1. About six million high-quality raw reads were obtained from the libraries. Selected reads from these libraries were mapped to the mice genome, representing 50.89% and 70.46% of the total reads. miRNAs comprised 49.45% and 26.31% of the total reads. The rest of the sequences were other types of RNA, including noncoding RNA, rRNA, scRNA, snRNA, snoRNA, srpRNA, and tRNA (Supplemental Table S1). The number of reads is shown in Supplemental Table S2. Using the selection criteria of (1) a fold change <0.7 for downregulation or >1.5 for upregulation after CLP and (2) at least one condition (with or without CLP) with more than 500 reads, 21 interesting miRNA targets were obtained.

### Expression of selected representative miRNAs in the exosomes

**Figure 3:**
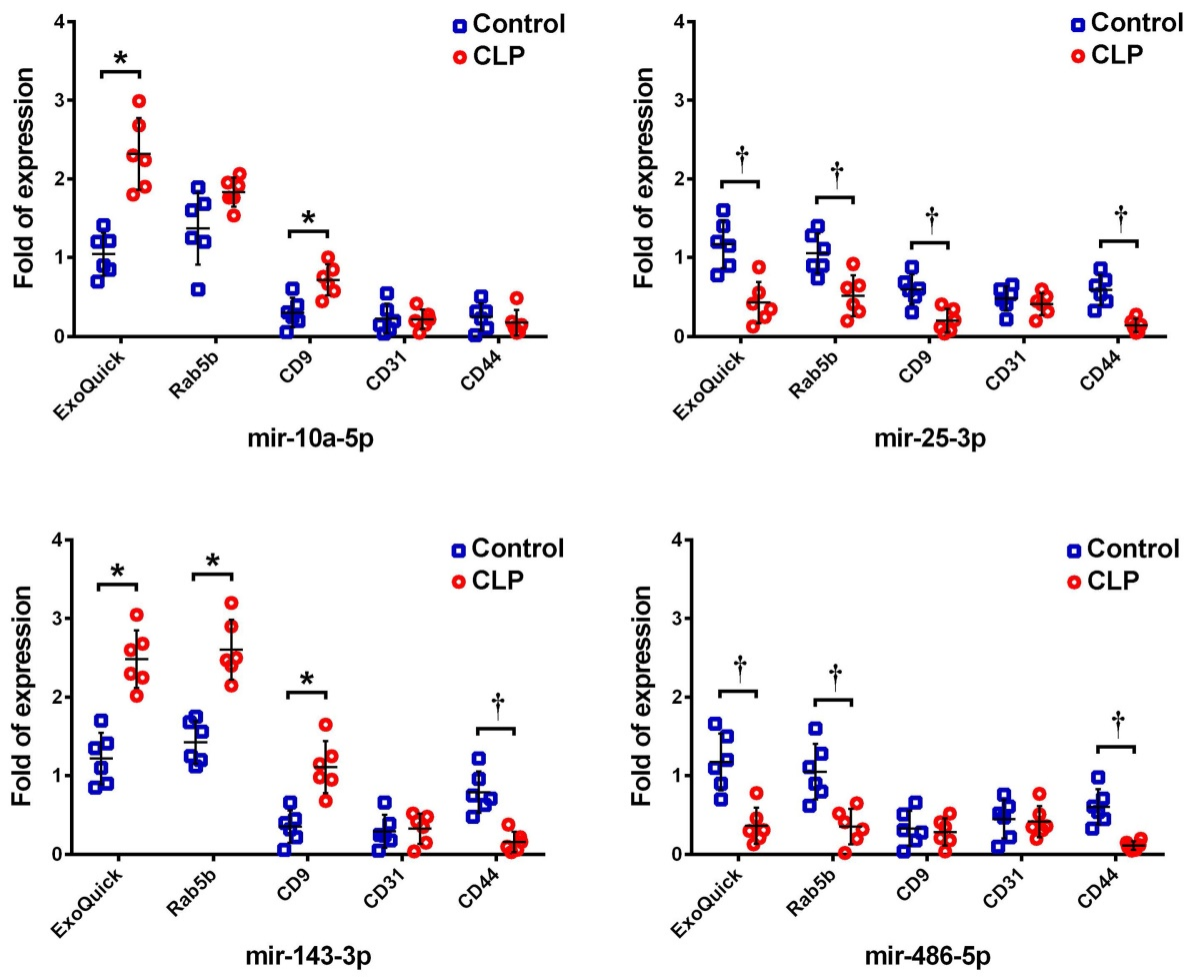
Expression of the four most abundant exosomal miRNAs (mmu-miR-486-5p, mmu-miR-10a-5p, mmu-miR-143-3p, and mmu-miR-25-3p) as detected by RT-qPCR in the total exosomes isolated by ExoQuick-TC and the subpopulations purified by Rab5b, CD9, CD31, and CD44 Exo-Flow Exosome Capture Kits following cecum ligation and perforation (CLP).

Among these 21 miRNAs, the four most abundant exosomal miRNAs (mmu-miR-486-5p, mmu-miR-10a-5p, mmu-miR-143-3p, and mmu-miR-25-3p) following CLP were selected as representative targets for validation by RT-qPCR. The expression levels of these miRNAs within the exosomes isolated by ExoQuick-TC and the subpopulations of exosomes purified using Rab5b, CD9, CD31, and CD44 Exo-Flow Exosome Capture Kits were measured both following CLP and in the sham controls. The expression levels of these four miRNAs in the exosomes isolated by ExoQuick-TC, as measured by RT-qPCR and NGS, were similar, showing upregulation of mmu-miR-10a-5p and mmu-miR-143-3p but downregulation of mmu-miR-25-3p and mmu-miR-486-5p following CLP against when compared the sham control (Figure 3). However, their expression levels in the antibody-captured exosome subpopulations varied. For Rab5b-captured exosomes, mmu-miR-143-3p was upregulated but mmu-miR-25-3p and mmu-miR-486-5p were downregulated following CLP when compared to the levels in the sham control. In CD9-captured exosomes, mmu-miR-10a-5p and mmu-miR-143-3p were upregulated but mmu-miR-25-3p was downregulated following CLP. In CD44-captured exosomes, mmu-miR-25-3p, mmu-miR-143-3p, and mmu-miR-486-5p were downregulated following CLP. In CD31-captured exosomes, none of these four miRNA targets differed significantly.

### Hierarchical clustering of the annotated functions

**Figure 4:**
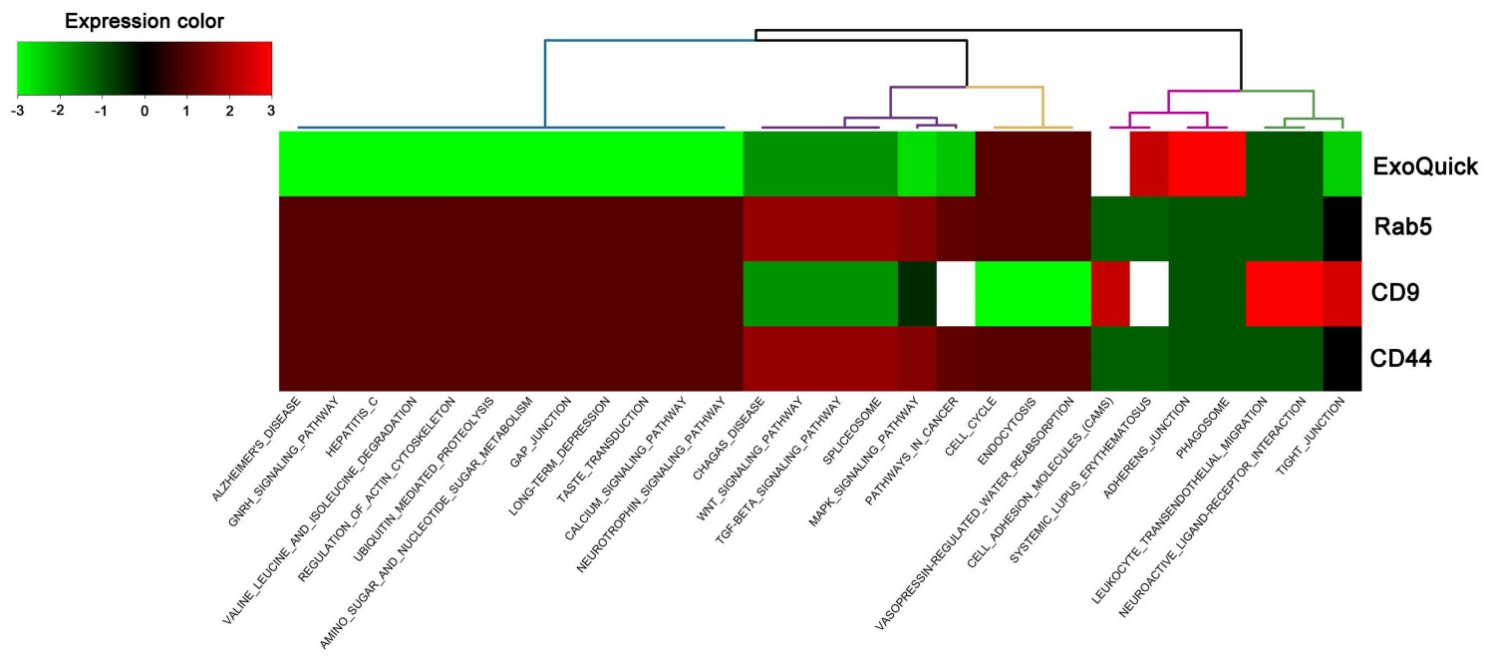
Hierarchical clustering of the annotated functions of predicted targets in miRSystem according to the differentially expressed miRNAs in total exosomes isolated by ExoQuick-TC and exosome subpopulations purified by Rab5b, CD9, and CD44 Exo-Flow Exosome Capture Kits from mice following cecum ligation and perforation (CLP).

The miRSystem was used for target prediction and functional annotation of the differentially expressed miRNAs within the total exosomes and exosome subpopulations captured via different surface markers. Hierarchical clustering of the annotated functions of the predicted targets of the differentially expressed miRNAs are shown in Figure 4, which revealed that the annotated functions of the antibody-captured exosomes differed from those of exosomes isolated via ExoQuick-TC. Although the patterns of annotated functions were similar between Rab5b- and CD44-captured exosomes, it should be noted that, following CLP, mmu-miR-143-3p was upregulated in Rab5b-captured exosomes but downregulated in CD44-captured exosomes. Furthermore, the annotated functions of the predicted targets could not be determined for CD31-captured exosomes, since there were no significant differences in the four selected miRNAs following CLP.

## Discussion

This study revealed that there are unique miRNA content patterns among exosome subpopulations purified via various exosomal markers. Our results are in accordance with those of other studies which revealed that the contents of EVs varied based the levels of protein markers like CD9, TSG101, and ALIX 24, 25. It has been reported that the miRNA concentrations varied greatly among exosomes purified via different isolation methods 15, 26. According to our study results, even when using the same method to isolate exosomes, purification via different exosomal markers collected different exosome subpopulations with distinct miRNA contents. Although it is generally believed that the surface proteins of exosomes, along with their molecular cargo, are a rich source of biomarkers for various pathological conditions 27, this study demonstrated the heterogeneity of circulating exosomes and implied the importance of stratifying exosome subpopulations when using circulating exosomes for biomarkers or investigating the functions of exosomes. In addition, this study also emphasized the necessity of using a consistent exosome marker across different samples when detecting biomarkers.

The isolation and purification of exosomes are still considered major scientific challenges 28, and there is no clear consensus on the single best method or even a standardized method for their isolation and purification 29, 30. Current methods used for the isolation of exosomes include ultracentrifugation 31, filtration 32, and immuno-affinity 33, 34, as well as various combinations thereof. Centrifugation can concentrate the exosomes in a sample but does not separate subpopulations, and thus exosomes obtained using this method only reflect the average properties of a heterogeneous exosome population. Filtration can also enrich or concentrate the exosomes of a targeted size population, but damage has been observed in the isolated exosome subpopulations, and the recovery efficiency and purity have been questioned 35. Furthermore, although the performance of ExoQuick is better than that of ultracentrifugation, ExoQuick-purified exosomes had with most contaminants among exosomes obtained from various isolation kits 36. Comparison of six commercial isolation methods (exoEasy, ExoQuick, Exo-spin, ME kit, ExoQuick Plus, and Exo-Flow) for serum exosomes showed that the cytokine concentrations were very different, depending on the purification kit used 37. In this study, the expression levels of four selected miRNAs in exosomes isolated by ExoQuick-TC were similar when measured by RT-qPCR and NGS. However, their expression levels varied among the antibody-captured exosome subpopulations. The patterns of these dysregulated miRNAs in exosomes captured by Rab5b or CD9 antibodies were more similar to those isolated by ExoQuick-TC than to those captured by CD44. However, for CD31-captured exosomes, none of these four miRNA targets showed a significant difference. Notably, the amount of exosome subpopulations isolated by the magnetic bead affinity method is very low, and generally less than 2 ug of exosomes could be harvested from 1 mL of blood of the mice. Therefore, this study is limited by the very low yield of the exosome subpopulations by magnetic bead affinity method to do experimental validation of the function of these dysregulated exosomal miRNAs.

Because circulating exosomes can be released by different types of cells, including circulating blood cells or other cells in close contact with the circulation, the population of exosomes present in the blood is very heterogeneous. It has been estimated that 80-90% of the circulating exosomes are released by platelets, lymphocytes, dendritic cells, and other immune cells 38, 39. Population heterogeneity presents one of the biggest challenges for exosome study. Discrimination of different populations of exosomes based on their surface antigens has been proposed. However, different reports have revealed that the surface marker tetraspanins CD9, CD63, and CD81 are not only abundant in exosomes but also in MVs 40, 41, complicating their usefulness as exosome biomarkers 40, 42. Given the differences in the composition and cellular origin of distinct subpopulations of exosomes, exosome studies based on their size and tetraspanin enrichment as the principal criteria should be considered more cautiously 17.

## Supplementary Material

Supplementary Table 1. Next-generation sequencing (NGS) analysis of miRNA expression in the circulating exosomes from mice following cecum ligation and perforation (CLP).
